# Shared Decision-Making in Veterinary Clinics: A Cross-Sectional Study Examining Effects on Appropriate Antibiotic Prescribing and Value-Driven Factors Influencing It

**DOI:** 10.3390/antibiotics14111139

**Published:** 2025-11-10

**Authors:** Huiling Guo, Lay See Ong, Zoe Jane-Lara Hildon, Timothy Chua, Boon Han Teo, Angela Chow

**Affiliations:** 1 Department of Epidemiology and Preventive Medicine, Office of Clinical Epidemiology, Analytics and Knowledge, Tan Tock Seng Hospital, Singapore 308433, Singapore; huiling.guo@nhghealth.com.sg (H.G.); laysee.ong@mohh.com.sg (L.S.O.); angela.chow@nhghealth.com.sg (A.C.); 2 Saw Swee Hock School of Public Health, National University of Singapore, and National University Health System, Singapore 117549, Singapore; 3 Beecroft Animal Specialist and Emergency Hospital, Singapore 119973, Singapore; timothy.chua@beecroft.com.sg; 4 Singapore Veterinary Association, Singapore 069110, Singapore; teo.boon.han@vettrustsingapore.com; 5 Lee Kong Chian School of Medicine, Nanyang Technological University, Singapore 308232, Singapore

**Keywords:** antimicrobial resistance, companion animals, veterinary clinics, antibiotic prescribing, shared decision-making

## Abstract

Background: Shared decision-making (SDM) can improve appropriate antibiotic prescribing in human primary care clinics, but little is known about it in the veterinary setting. This study examines the association between SDM and antibiotic prescribing for cats and dogs in veterinary clinics, then determines the key factors influencing SDM through a value-driven approach. Methods: This cross-sectional study surveyed 41 veterinarians practicing in Singapore veterinary clinics. Appropriate antibiotic prescribing was defined as concordance with local antibiotic guidelines for companion animals, while an adapted SDM-Q-9-Doc scale measured SDM on antibiotic prescribing with pet owners. Questions were developed to measure constructs of the VALUE model and factor analysis was conducted to identify latent factors. Multivariable logistic and linear regressions were performed to assess the association between SDM and antibiotic prescribing, and determinants of SDM, respectively. Results: Antibiotics were most inappropriately prescribed for cats/dogs with ear infections (cocci observed) (63%), and periodontal disease (59%). Engaging owners in SDM (AOR 1.31; 95% CI 1.05, 1.62; *p* = 0.014) was positively associated with appropriate antibiotic prescribing for cats/dogs with periodontal disease. After adjusting for age, gender, clinic role and practice type, veterinarians with a higher overall VALUE score (β = 0.14; 95% CI 0.02, 0.26; *p* = 0.021) were more likely to engage in SDM with owners on antibiotic prescribing. Conclusions: SDM with pet owners improves appropriate antibiotic prescribing for cats and dogs, though effects vary by clinical scenario. Aligning AMR-related values between veterinary clinics and veterinarians to enable effective communication with pet owners can promote SDM and subsequently drive appropriate antibiotic prescribing for pet cats and dogs.

## 1. Introduction

In 2021, antimicrobial resistance (AMR) claimed the lives of 4.71 million people globally [1]. AMR will continue to threaten global health, causing up to 10 million deaths annually by 2050 [2]. Yet, tackling this silent pandemic of AMR extends far beyond the human population. The interconnectedness and interdependency between the human health, animal health and environmental sectors—also known as One Health—calls for concerted efforts in these domains to slow down the progression of AMR [3,4].

Although misuse and overuse of antibiotics constitute the primary drivers of AMR [5], research investigating antibiotic use in veterinary settings for companion animals, or pets, remains underrepresented relative to studies conducted in human primary care [6], human tertiary care [7,8], and animal farming sectors [9]. This is despite clear evidence of AMR transmission between pets and their owners [10], and a growing pet ownership globally [11,12,13].

As conceptualised by Hopman et al. [14], owner-, pet-, treatment-, and context-related determinants influence veterinarian-related factors, which in turn determine antibiotic use in companion animals. However, achieving owner satisfaction, with aims to sustain clientele pool to maintain business viability for their veterinary clinic, often impacts how antibiotic decisions are made by veterinarians [15,16]. It is not uncommon that veterinarians would misinterpret that owners are anxious and demand a quick and affordable treatment solution, including antibiotics, for their companion animals [15], not knowing that these owners are likely to agree with them even if antibiotics are unwarranted [15,17].

Similarly, shared decision-making (SDM)—a mechanism proven to be effective in reducing inappropriate antibiotic prescribing in human primary care [18]—is primarily employed for general animal care [19,20], instead of antibiotic prescribing for companion animals, to achieve good owner satisfaction [21,22]. However, in contrast to human primary care, little is known about the effect of SDM on antibiotic prescribing for companion animals, and how SDM can be promoted in veterinary clinics for the purpose of antibiotic stewardship.

In the recent literature, there is a paradigm shift towards value-conscious and systemic approaches that recognise the need for alignment of organisational priorities with collective responsibilities on antibiotic stewardship to tackle AMR [23]. As encapsulated by a VALUE model [24], alignment of AMR-related values between organisations (human primary care clinic or veterinary clinic) and prescribers (primary care doctor or veterinarian) enables effective consumer (patients or pet owners) communication and SDM engagement, driving appropriate antibiotic prescribing [16,24]. Hence, in this study, we aim to first examine the association between SDM and antibiotic prescribing for companion animals in veterinary clinics, and, using a value-driven approach, determine the key factors influencing SDM based on constructs mapped onto the VALUE model. The VALUE model was developed in human primary care clinics [24] and successfully transferred to the veterinary setting for application [16].

## 2. Results

### 2.1. Basic Characteristics of Survey Respondents

During the study period, 41 survey responses were collected, with their basic characteristics described under Table 1. Overall, veterinarians who responded to the survey were young, with a mean age of 37.0 ± 8.2 years old and a mean of 10.4 ± 8.1 years since attaining basic veterinary degree. Most were females (71%), from solo practices (46%), working full-time (83%), and identified themselves as employees in their clinics (49%).

### 2.2. Self-Reported Antibiotic Prescribing Behaviours

An equal proportion of veterinarians (76%) reportedly saw up to 10 cats and up to 10 dogs, respectively, in a typical clinic day. More than one-quarter (29%) of the veterinarians prescribed antibiotics to ≤10% of the cats seen in a typical clinic day, 41% to 11–20% of cats seen, 17% to 21–30% of cats seen, and 12% to >30% of cats seen. On the other hand, nearly a quarter (24%) of the veterinarians prescribed antibiotics to ≤10% of the dogs seen in a typical clinic day, 39% to 11–20% of dogs seen, 27% to 21–30% of dogs seen, and 10% to >30% of dogs seen.

**Figure 1:**
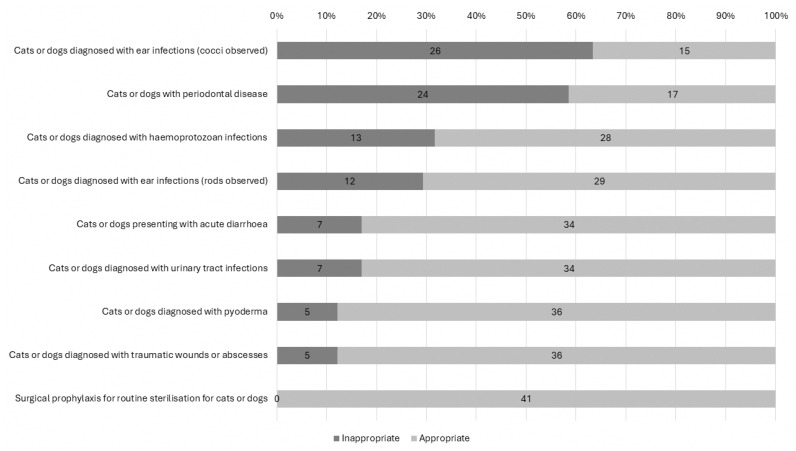
Proportion of inappropriate antibiotic prescribing, i.e., not in concordance with the local prescribing guidelines, (based on initial antibiotic choice) for various scenarios presented at the veterinary clinic (N = 41). Note: Exact counts are represented within the graphs.

Out of the nine clinical scenarios for cats and dogs presented in Figure 1, ear infections with cocci observed (N = 26; 63%) and periodontal disease (N = 24; 59%) were conditions with more than half of the respondents reportedly prescribing antibiotics inappropriately, i.e., not in concordance with the local antibiotic prescribing guidelines. Overall, more than half of the veterinarians (N = 24; 59%) prescribed antibiotics appropriately for ≥7 out of the 9 clinical scenarios, and they tended to be younger (34.1 ± 6.4 years old vs. 41.1 ± 8.8 years old; *p* = 0.005).

### 2.3. Shared Decision-Making and Appropriate Antibiotic Prescribing

**Figure 2:**
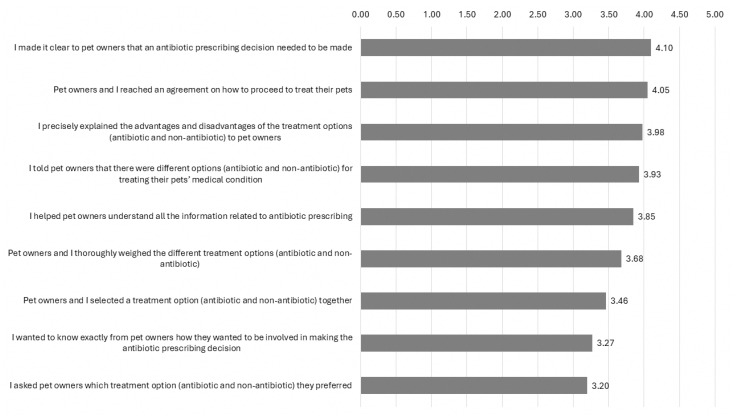
Mean score of responses on statements regarding shared decision-making with owners of pet cats and dogs on antibiotic prescribing, adapted from SDM-Q-9-Doc (N = 41).

Veterinarians were most likely to clarify with owners that an antibiotic prescribing decision needs to be made (mean score: 4.10 [SD 0.89]), while they were least likely to ask owners on which treatment option (antibiotic or non-antibiotic) they would prefer (mean score: 3.20 [SD 1.14]) (Figure 2).

**Figure 3:**
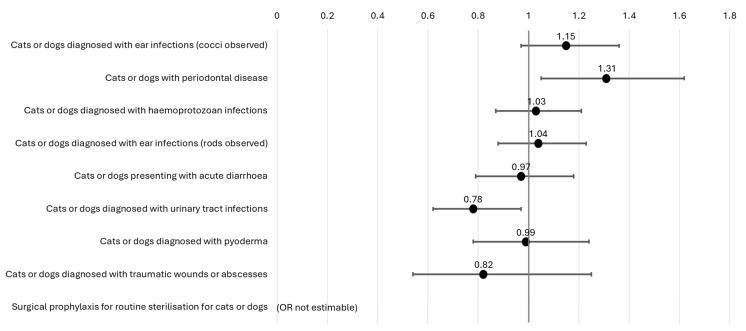
Odds ratios, adjusted for age, gender, practice type and clinic role, showing relationship between shared decision-making and appropriate antibiotic prescribing across various scenarios presented at the veterinary clinic (N = 41).

Adjusted for age, gender, practice type and clinic role, veterinarians who engaged in SDM with owners on antibiotic prescribing (AOR 1.31; 95% CI 1.05, 1.62; *p* = 0.014) were more likely to prescribe antibiotics appropriately for cats and dogs with periodontal disease (Figure 3). On the other hand, adjusted for age, gender, practice type and clinic role, veterinarians who engaged in SDM with owners on antibiotic prescribing (AOR 0.78; 95% CI 0.62, 0.97; *p* = 0.028) were less likely to prescribe antibiotics appropriately for cats and dogs with urinary tract infections. Any association with SDM was not observed in the top clinical scenario with highest proportion of antibiotics inappropriately prescribed, i.e., cats and dogs with ear infections (cocci observed).

However, upon exploring its association with individual SDMQ-9-Doc items, veterinarians who asked owners on which treatment option (antibiotic or non-antibiotic) they would prefer (AOR 2.92; 95% CI 1.18, 7.20; *p* = 0.020) were more likely to prescribe antibiotic appropriately for cats and dogs with ear infections (cocci observed), when adjusted for age, gender and clinic role.

### 2.4. Factors Influencing Shared Decision-Making on Antibiotic Prescribing

The number of items measuring the constructs of the VALUE model [24] were reduced from 65 to 35, classified under nine factors: **V**alues (three factors—PV, OV1 and OV2), **A**lignment (two factors—A1 and A2), **L**iaison (two factors—L1 and L2), **U**se of monitoring and **E**valuation data (two factors—UE1 and UE2) (Table 2). Each factor has a high internal consistency, with a Cronbach’s alpha value of >0.8.

The combined VALUE approaches, as reflected by the VALUE composite score, (Model 2: β = 0.14; 95% CI 0.02, 0.26; *p* = 0.021) is positively associated with engaging in SDM with owners on antibiotic prescribing, after adjusting for age, gender, clinic role and practice type. On the other hand, when individual factors were investigated independently, veterinarians who perceived the organisation’s value of prioritising pet owner satisfaction over appropriate antibiotic prescribing (crude β = 2.69; 95% CI 0.35, 5.03; *p* = 0.026), those who had the capacity to build relationships and communicate with owners on antibiotic prescribing (crude β = 2.83; 95% CI 0.98, 4.67; *p* = 0.004) and those who believed in collaborative communication and shared decision-making with owners on antibiotic prescribing (crude β = 3.61; 95% CI 1.70, 5.51; *p* < 0.001) were more likely to engage in SDM with owners on antibiotic prescribing (Table 3). In contrast, veterinarians who perceived influence of national-level audits on antibiotic utilisation amongst local veterinary clinics (crude β = −2.17; 95% CI −4.32, −0.02; *p* = 0.048) were less likely to engage in SDM with owners on antibiotic prescribing, with a borderline statistical significance. However, after adjusting for age, gender, clinic role and practice type under Model 1, these constructs failed to sustain a statistically significant association with SDM.

## 3. Discussion

This study has provided valuable insights into SDM and appropriate antibiotic prescribing for pet cats and dogs in local veterinary clinics. In the local veterinary clinics, we observed that more than half of veterinarians showed good alignment of antibiotic prescribing practices with guideline recommendations. This is not unexpected as the first set of local prescribing guidelines published in 2021 was developed in close consultation with practicing veterinarians in Singapore [25]. Furthermore, veterinarians had desired for this local practice guidance [16]. However, despite being a good resource, it was previously opined by veterinarians that the guidelines may not be fully relevant for clinical practice due to their non-applicability for complex cases often seen by them [16], calling for regular review to constantly update and improve the recommendations to enhance guideline compliance.

For national guidelines to be successfully implemented and adopted in the veterinary clinic setting, clinic dynamics should not be disregarded. Clinic-specific antibiotic prescribing practices are much more emphasised and followed by fellow veterinarians practicing in the same clinic [16]. These practices are often set by key decision-making veterinarians [16], who tend to be older and less aligned with recommendations by the guidelines as found in this study. Therefore, beyond expert opinions [26], authorities should consider involving practicing veterinarians—especially those who are key decision-makers in their clinics—in joining the panel to provide their inputs to improve the guidelines with acceptable and practicable recommendations, which will be better adopted by the wider veterinary community.

Concurring with what was reported by pet cat and dog owners [17], SDM often involves the clarification between veterinarians and owners on the need to make an antibiotic prescribing decision but is seldom related to asking owners to choose their preferred treatment option (antibiotic or non-antibiotic) for their companion animal. Rather than antibiotic choice, discussions are more likely to surround antibiotic administration instead [17], to enable appropriate antibiotic use by owners through overcoming feeding difficulties to achieve better medication adherence [14,17,27].

Through our study, we found that SDM with owners can influence appropriate antibiotic prescribing for pet cats and dogs with varying effects across different clinical scenarios. A positive association was found between SDM and appropriate antibiotic prescribing for periodontal disease, but the converse was evident for urinary tract infections. Apart from that, there was no significant relationship between SDM and other clinical scenarios. While this variability could be attributed to a lack of statistical power due to a small sample size, these observations had shed light on how SDM can play an important role in antibiotic stewardship within the veterinary clinic setting, besides general animal care [19,20].

As recommended by the local guidelines [25], topical treatment is sufficient, and systemic antibiotics are only warranted for severe periodontal diseases. However, dental conditions could often limit nutrient intake in companion animals and necessitate dietary changes due to chewing difficulties. This can increase owner anxiety and emotional stress, potentially leading to an unnecessary demand for antibiotics to be prescribed as a perceived quick solution [28]. Through SDM, these concerns can be addressed and managed [17]. On the other hand, antibiotic prescribing decisions for urinary tract infections should be guided by culture and sensitivity testing [25]. Involving owners in these decisions that require veterinary expertise could lead to inappropriate antibiotic choice being made [17], and hence the negative association observed between SDM and appropriate antibiotic prescribing for urinary tract infections in this study. In general, the effectiveness of SDM in promoting appropriate antibiotic prescribing appears to be condition-specific in veterinary clinics and should be examined separately in future studies.

Beliefs in collaborative communication and shared decision-making with owners on antibiotic prescribing, as motivated by the belief in honouring an owner’s rights to make decisions for their companion animals [17], can promote SDM. While SDM is not a mandatory practice in veterinary clinics [17], engagement of SDM with owners on antibiotic prescribing is often motivated by the perception that their clinic prioritises owner satisfaction—ironically, over appropriate antibiotic prescribing—which is likely driven by the emphasis on sustaining a clientele pool to maintain business viability for their clinic [15,16]. These observations further confirm findings from a prior qualitative study [16] that established antibiotic stewardship practices, such as effective communications with owners and fostering trusting owner–veterinarian relationships, are normally conceptualised as methods to improve owner experience rather than being acknowledged for their role in promoting antibiotic stewardship [29]. Even though there is a lack of statistical power to support the current findings with confidence, both past [16,17] and present studies shed light on a unique antibiotic stewardship approach in veterinary clinics that could encourage the prioritisation of owner experience and satisfaction for an indirect benefit of driving effective communication with owners to promote SDM on antibiotic prescribing. As SDM is a two-way communication process, encouraging veterinarians to take initiative in investigating owner’s feeding concerns and delivering training and advice to owners on antibiotic administration methods for their companion animals in specific will better involve pet owners in discussions related to antibiotic prescribing [17]. Better time and resource planning to allow veterinarians to have the capacity to build relationships and communicate with owners on antibiotic prescribing can encourage the process of SDM as well.

Based on our knowledge, this is the first-ever study conducted amongst veterinarians practicing in veterinary clinics in Singapore that assess the rate of inappropriate antibiotic prescribing for pet cats and dogs, explore the relationship between SDM and antibiotic prescribing for pet cats and dogs, and examine the determinants that can promote SDM between veterinarians and owners to discuss antibiotic prescribing matters. The use of the SDM-Q-9-Doc scale [28] in this study enables the comparison of findings with a previous study conducted with pet owners using the SDM-Q-9 scale [17] to measure agreement in the occurrence of SDM items reported.

Most importantly, the development of items for a structured assessment of constructs from the VALUE model [24] has strengthened the survey instrument’s theoretical grounding and content validity, and successfully quantified the themes emerged from a preceding exploratory qualitative study [16]. This study further proves the theoretical pathway, as demonstrated by the VALUE model, that AMR-related value alignment between veterinary clinics and veterinarians facilitates effective communications with pet owners, thereby promoting SDM and appropriate antibiotic prescribing [24]. On top of that, a high internal consistency observed for each of the nine reduced factors adds reliability to the 35-item VALUE scale and confidence to the application of the scale to a wider veterinarian population in the future.

Nonetheless, this study is limited by a small sample size which may have compromised the statistical power to detect meaningful associations between variables, especially between appropriate antibiotic prescribing, SDM and the constructs of VALUE model. Even though the survey is anonymous and would therefore reduce social desirability bias, this study is subjected to potential selection bias in relation to volunteer bias. The findings may also not be generalisable to other veterinary settings.

## 4. Materials and Methods

### 4.1. Study Design and Study Population

This is a cross-sectional study. Veterinarians practicing in veterinary clinics in Singapore were invited to take part in a self-administered online survey between April 2024 and May 2025. During the study period, it was estimated that there were about 600 licenced veterinarians in Singapore, including those not practicing in veterinary clinics [30,31].

Only veterinarians licenced under Singapore’s Animal & Veterinary Service and practicing in a local veterinary clinic were included in this study. Emails were first sent by the study team to all veterinary clinics in Singapore (i.e., 107 clinics at the point of recruitment) via their respective key contact personnel to invite all veterinarians who were practicing in their premises to take part in the survey. This was carried out to ensure that all eligible veterinarians were reached to reduce selection bias. Additional efforts were later made to boost recruitment through physical visits to veterinary clinics and through recruitment booths set up at two major veterinary conferences held locally.

### 4.2. Survey Instrument

The survey instrument was designed with close-ended questions. *Self-reported antibiotic prescribing behaviours* were assessed through the proportions of pet cats and dogs seen in a typical clinic day which were prescribed antibiotics, respectively. *Appropriate antibiotic prescribing* for different clinical scenarios presented in the survey was defined as concordance with the antibiotics recommended by a set of antibiotic prescribing guidelines jointly released by the Animal and Veterinary Service and Singapore Veterinary Association, Singapore’s veterinary authority and professional body, respectively [25]. *SDM* was assessed using a composite score of nine items from the Scholl et al.’s SDM-Q-9-Doc scale [32], presented in a 5-point Likert scale (1—strongly disagree to 5—strongly agree) and reworded to fit the veterinary context. Sixty-five items measuring the constructs of the *VALUE model* [24]—**V**alues, **A**lignment, **L**iaison, **U**se of monitoring and **E**valuation data—were developed by HG and LSO, and finalised through close discussions with ZJH. Further socio-demographic information was collected, and the survey was available in English language only.

### 4.3. Data Analysis

Categorical and dichotomised variables were presented as proportions, while continuous variables were presented as means. Exploratory factor analysis, using principal factors method with varimax rotation was conducted separately for items within each VALUE model construct to identify the optimal factor structure. Items with unrotated factor loading ≥ 0.7 were retained for further analyses, and some constructs were split into two separate latent factors based on empirical data. Factor-specific analyses were then performed for each of the latent factors, with items demonstrating factor loadings < 0.7 retained based on internal consistency evaluated using Cronbach’s alpha coefficients. Correlation between these latent factors were examined, and a composite VALUE score was calculated from the retained items, with negatively phrased items recoded before summation. Multivariable logistic regression was performed to examine the association between SDM and appropriate antibiotic prescribing, and multivariable linear regression was performed to determine the independent factors associated with veterinarian’s engagement in SDM on antibiotic prescribing with owners. Robust standard errors using the HC3 estimator were applied for linear regression models to account for heteroskedasticity and improve small-sample inference properties. Covariates were selected through assessing the Akaike information criteria, Bayesian information criteria and likelihood ratios, and included in the final regression model to adjust for potential confounding. Statistical significance was benchmarked as *p*-value < 0.05 and statistical analyses were conducted in Stata version 18.0 (StataCorp LLC, College Station, TX, USA).

## 5. Conclusions

Poor alignment of antibiotic practices with local guideline recommendations for pet cats and dogs is observed. Engaging in SDM with pet owners can improve appropriate antibiotic prescribing for pet cats and dogs with periodontal disease. Aligning AMR-related values between veterinary clinics and veterinarians to enable effective communication with pet owners can promote SDM and subsequently drive appropriate antibiotic prescribing for pet cats and dogs.

## Figures and Tables

**Table 1:** Basic characteristics of survey respondents.

Characteristic	Total (N = 41)
***Age, in years***	
Mean (SD)	37.0 (8.2)
***Gender, N (%)***	
Female	29 (71)
***Employment status, N (%)***	
Full-time permanent	34 (83)
Part-time permanent	3 (7)
Locum	4 (10)
***Practice type, N (%)***	
Solo practice with only 1 clinic under its name	19 (46)
Small group practice with 2 clinics under its name	11 (27)
Large chain practice with more than 2 clinics under its name	7 (17)
Hospital or referral centre	4 (10)
***Country of basic veterinary degree, N (%)***	
Australia	27 (66)
United Kingdom	11 (27)
New Zealand	3 (7)
***Years of practice since attaining basic veterinary degree, in years***	
Mean (SD)	10.4 (8.1)
***Years of practice in Singapore, in years***	
Mean (SD)	9.7 (7.7)
***Years of practice in current clinic, in years***	
Mean (SD)	6.0 (6.8)
***Clinic role, N (%)***	
Manager or key decision-maker	17 (41)
Employee	20 (49)
Locum	4 (10)

SD: Standard deviation.

**Table 2:** A total of 35 items measuring constructs under the VALUE model, and their respective Cronbach’s alpha.

VALUE Model Construct	Item	Factor Loading	Cronbach’s Alpha
**Values**—Own internalised values and beliefs pertaining to antibiotic prescribing and antimicrobial resistance in the veterinary setting **(PV)**	I believe that by prescribing antibiotics appropriately…	0.7783	0.9082
	It is important for me to comply with antibiotic prescribing practice…	0.7972	
	I believe that I can make an impact on AMR…	0.8919	
	To me, AMR…	0.8901	
**Values**—Organisational (i.e., within your veterinary clinic) culture on appropriate antibiotic prescribing **(OV1)**	My clinic has clear goals…	0.7419	0.8127
	Prescribing antibiotics appropriately…	0.7065	
	My clinic devotes resources…	0.7926	
**Values**—Perceived organisation’s value of prioritising owner satisfaction over appropriate antibiotic prescribing **(OV2)**	Between maintaining customer satisfaction and prescribing antibiotics appropriately…	0.7610	0.8086
	My clinic will prioritise pet owners’ satisfaction over prescribing antibiotics appropriately…	0.7610	
**Alignment**—Measure of perceived person–organisation alignment with regard to values on antibiotic prescribing and antimicrobial resistance **(A1)**	My clinic and I are aligned…	0.9275	0.9735
	There is a good match between how I want to make my antibiotic prescribing decisions…	0.9197	
	My personal values with regard to antibiotic prescribing and AMR…	0.9587	
	My clinic’s values and culture provide a good fit…	0.9580	
	There is great similarity between my values with regard to antibiotic prescribing and AMR…	0.8466	
	The things that I value with regard to antibiotic prescribing…	0.9742	
**Alignment**—Perceived degree of individuals influencing organisational antimicrobial resistance values and antibiotic prescribing practices **(A2)**	Regardless of designation, my co-workers and I are able to share views…	0.7524	0.9199
	My clinic is willing to listen to its employees…	0.8866	
	My clinic’s management is open to my suggestions…	0.9047	
	My clinic’s protocols on antibiotic prescribing practices…	0.7651	
	It is possible for me to influence how my clinic…	0.8754	
**Liaison**—Capacity to build relationships and communicate with owners on antibiotic prescribing **(L1)**	I am able to communicate with pet owners on antibiotic prescribing decisions…	0.7896	0.8255
	Lack of good relationships with pet owners prevents…	0.6668	
	I am able to gain the trust of pet owners…	0.7715	
	It is easy for me to identify the potential needs…	0.6875	
**Liaison**—Beliefs in collaborative communication and shared decision-making with owners on antibiotic prescribing **(L2)**	I do not believe that veterinarians should engage in shared decision-making…	−0.7692	0.8416
	Finding out the potential concerns/issues faced by pet owners…	0.7120	
	It is important to ensure that pets’ antibiotic treatment decisions…	0.7439	
	A successful communication outcome for pets’ antibiotic treatment…	0.8218	
**Use of monitoring and Evaluation data**—Perceived influence of national-level audits on antibiotic utilisation among local veterinary clinics **(UE1)**	National-level audits (e.g., reporting number / appropriateness of antibiotics prescribed, etc.) compared to other veterinary clinics are helpful…	0.7954	0.8659
	National-level audits are not likely to provide good indicators…	−0.7606	
	Having data comparing veterinary practices nationally will encourage…	0.8185	
	National-level audits will not be useful in ensuring…	−0.7767	
**Use of monitoring and Evaluation data**—Perceived usefulness of audit and feedback mechanisms on individual antibiotic prescribing practices within the veterinary clinic **(UE2)**	Audit data are helpful to indicate…	0.8171	0.8448
	Audit data are instrumental to improve…	0.8332	
	Audit data will enable me to…	0.6850	

**Table 3:** Univariate and multivariable analyses on factors influencing shared decision-making on appropriate antibiotic prescribing (N = 41).

Variables	Univariate Analysis (N = 41)		Multivariable Analysis Model 1 (N = 41)		Multivariable Analysis Model 2 (N = 41)	
	Coefficient (95% CI)	*p*-Value *	Coefficient (95% CI)	*p*-Value *	Coefficient (95% CI)	*p*-Value *
**PV:** Own internalised values and beliefs pertaining to antibiotic prescribing and antimicrobial resistance in the veterinary setting	1.42 (−0.31, 3.15)	0.106	0.12 (−3.19, 3.43)	0.941	-	-
**OV1:** Organisational (i.e., within your veterinary clinic) culture on antibiotic prescribing	1.20 (−1.17, 3.58)	0.312	0.58 (−1.95, 3.11)	0.641	-	-
**OV2:** Perceived organisation’s value of prioritising owner satisfaction over appropriate antibiotic prescribing	2.69 (0.35, 5.03)	**0.026**	2.88 (−0.12, 5.89)	0.059	-	-
**A1:** Measure of perceived person–organisation alignment with regard to values on antibiotic prescribing and antimicrobial resistance	1.75 (−0.23, 3.72)	0.081	0.38 (−2.77, 3.53)	0.806	-	-
**A2:** Perceived degree of individuals influencing organisational antimicrobial resistance values and antibiotic prescribing practices	1.45 (−0.15, 3.05)	0.074	−1.01 (−3.67, 1.64)	0.441	-	-
**L1:** Capacity to build relationships and communicate with owners on antibiotic prescribing	2.83 (0.98, 4.67)	**0.004**	1.50 (−2.82, 5.81)	0.483	-	-
**L2:** Beliefs in collaborative communication and shared decision-making with owners on antibiotic prescribing	3.61 (1.70, 5.51)	**<0.001**	2.33 (−0.62, 5.28)	0.117	-	-
**UE1:** Perceived influence of national-level audits on antibiotic utilisation among local veterinary clinics	−2.17 (−4.32, −0.02)	**0.048**	−0.63 (−3.28, 2.01)	0.627	-	-
**UE2:** Perceived usefulness of audit and feedback mechanisms on individual antibiotic prescribing practices within the veterinary clinic	−2.08 (−4.29, 0.13)	0.064	−0.27 (−3.15, 2.61)	0.849	-	-
**VALUE composite score**	0.17 (0.05, 0.28)	**0.007**	-	-	0.14 (0.02, 0.26)	**0.021**
**Age**	0.07 (−0.28, 0.42)	0.704	−0.23 (−0.47, 0.01)	0.057	0.01 (−0.28, 0.30)	0.940
**Gender**						
Male	Ref	-	Ref	-	Ref	-
Female	−3.87 (−7.14, −0.61)	**0.021**	−1.81 (−5.69, 2.06)	0.345	−2.63 (−6.80, 1.54)	0.209
**Clinic role**						
Non-manager or key decision-maker	Ref	-	Ref	-	Ref	-
Manager or key decision-maker	1.74 (−1.76, 5.23)	0.321	2.43 (−1.88, 6.74)	0.257	−1.63 (−5.66, 2.40)	0.417
**Practice type**						
Large chain practice with more than 2 clinics under its name or hospital or referral centre	Ref	-	Ref	-	Ref	-
Solo practice with only 1 clinic under its name or small group practice with 2 clinics under its name	5.17 (1.38, 8.96)	**0.009**	2.03 (−2.05, 6.12)	0.316	4.47 (0.61, 8.34)	**0.025**

* Bold values indicate statistical significance of *p* < 0.05. CI: Confidence interval.

## Data Availability

The datasets used and/or analysed during the current study are available from the corresponding author on reasonable request.

## References

[1] GBD 2021 Antimicrobial Resistance Collaborators (2024). Global burden of bacterial antimicrobial resistance 1990–2021: A systematic analysis with forecasts to 2050. Lancet.

[2] O’Neill J. (2016). Tackling Drug-Resistance Infections Globally: Final Report and Recommendations. The Review on Antimicrobial Resistance. https://amr-review.org/sites/default/files/160518_Final%20paper_with%20cover.pdf.

[3] Pattis I., Weaver L., Burgess S., Ussher J.E., Dyet K. (2022). Antimicrobial Resistance in New Zealand-A One Health Perspective. Antibiotics.

[4] World Health Organization (2015). Global Action Plan on Antimicrobial Resistance. https://iris.who.int/server/api/core/bitstreams/1a487887-e162-46a0-8aef-802907c66070/content.

[5] World Health Organization Antimicrobial Resistance. 21 November 2023. https://www.who.int/news-room/fact-sheets/detail/antimicrobial-resistance.

[6] Kasse G.E., Humphries J., Cosh S.M., Islam M.S. (2024). Factors contributing to the variation in antibiotic prescribing among primary health care physicians: A systematic review. BMC Prim. Care..

[7] Wojcik G., Ring N., McCulloch C., Willis D.S., Williams B., Kydonaki K. (2021). Understanding the complexities of antibiotic prescribing behaviour in acute hospitals: A systematic review and meta-ethnography. Arch. Public Health.

[8] Reali S., Kwang Y.C., Cho J.G., Alffenaar J.W., Aslani P. (2025). Factors influencing physicians’ antimicrobial prescribing decisions: A systematic review of qualitative studies. Br. J. Clin. Pharmacol..

[9] McKernan C., Benson T., Farrell S., Dean M. (2021). Antimicrobial use in agriculture: Critical review of the factors influencing behaviour. JAC Antimicrob Resist..

[10] Caddey B., Fisher S., Barkema H.W., Nobrega D.B. (2025). Companions in antimicrobial resistance: Examining transmission of common antimicrobial-resistant organisms between people and their dogs, cats, and horses. Clin. Microbiol. Rev..

[11] AVMA News “Pet Population Continues to Increase While Pet Spending Declines”. 10 October 2024. https://www.avma.org/news/pet-population-continues-increase-while-pet-spending-declines.

[12] Animal Medicines Australia (2022). Pets in Australia: A National Survey of Pets and People.

[13] CNA “The Big Read: ‘Part of the Family’—The Rising Status of Pets Among Households and What It Means for Society”. 19 June 2023. https://www.channelnewsasia.com/today/big-read/pets-part-family-rising-status-big-read-3569031.

[14] Hopman N.E.M., Hulscher M.E.J.L., Graveland H., Speksnijder D.C., Wagenaar J.A., Broens E.M. (2018). Factors influencing antimicrobial prescribing by Dutch companion animal veterinarians: A qualitative study. Prev. Vet. Med..

[15] Tompson A.C., Mateus A.L.P., Brodbelt D.C., Chandler C.I.R. (2021). Understanding Antibiotic Use in Companion Animals: A Literature Review Identifying Avenues for Future Efforts. Front. Vet. Sci..

[16] Guo H., Hildon Z.J., Wong L.H., Chua T., Teo B.H., Chow A. (2025). The VALUE of antibiotic stewardship for companion animals: Understanding appropriate antibiotic prescribing for pet cats and dogs in veterinary clinics in Singapore. One Health.

[17] Guo H., Hildon Z.J., Chua T., Teo B.H., Chow A. (2025). An exploratory mixed methods study on shared decision-making and antibiotic prescribing for pet cats and dogs in Singapore veterinary clinics. Sci. Rep..

[18] Tonkin-Crine S.K., Wang K., van Hecke O., Roberts N.W., McCullough A., Hansen M.P., Butler C.C., Del Mar C.B. (2017). Clinician-targeted interventions to influence antibiotic prescribing behaviour for acute respiratory infections in primary care: An overview of systematic reviews. Cochrane Database Syst Rev..

[19] Englar R.E. (2023). Recasting the gold standard—Part I of II: Delineating healthcare options across a continuum of care. J. Feline Med. Surg..

[20] Englar R.E. (2023). Recasting the gold standard—Part II of II: Communicating healthcare options along a continuum of care. J. Feline Med. Surg..

[21] Janke N., Coe J.B., Bernardo T.M., Dewey C.E., Stone E.A. (2021). Pet owners’ and veterinarians’ perceptions of information exchange and clinical decision-making in companion animal practice. PLoS ONE.

[22] Ito Y., Ishikawa H., Suzuki A., Kato M. (2022). The relationship between evaluation of shared decision-making by pet owners and veterinarians and satisfaction with veterinary consultations. BMC Vet. Res..

[23] Broom A., Kenny K., Prainsack B., Broom J. (2021). Antimicrobial resistance as a problem of values? Views from three continents. Crit. Public Health.

[24] Guo H., Hildon Z.J., Loh V.W.K., Sundram M., Ibrahim M.A.B., Tang W.E., Chow A. (2021). Exploring antibiotic prescribing in public and private primary care settings in Singapore: A qualitative analysis informing theory and evidence-based planning for value-driven intervention design. BMC Fam. Pract..

[25] National Parks Board, Animal and Veterinary Service, Singapore Veterinary Association *Guidelines for the Prudent Use of Antimicrobials in Companion Animals*; 22 November 2021. https://www.nparks.gov.sg/-/media/avs/amr/amr-pug-w_o-annex-(updated).pdf?la=en&hash=B2F939FD47FA6DDFF0036F9A5FD097E2A5C7F10F.

[26] Allerton F., Jeffery N. (2020). Prescription rebellion: Reduction of antibiotic use by small animal veterinarians. J. Small Anim. Pract..

[27] Burke S., Black V., Sánchez-Vizcaíno F., Radford A., Hibbert A., Tasker S. (2017). Use of cefovecin in a UK population of cats attending first-opinion practices as recorded in electronic health records. J. Feline Med. Surg..

[28] Dickson A., Smith M., Smith F., Park J., King C., Currie K., Langdridge D., Davis M., Flowers P. (2019). Understanding the relationship between pet owners and their companion animals as a key context for antimicrobial resistance-related behaviours: An interpretative phenomenological analysis. Health Psychol. Behav. Med..

[29] Currie K., King C., Nuttall T., Smith M., Flowers P. (2018). Expert consensus regarding drivers of antimicrobial stewardship in companion animal veterinary practice: A Delphi study. Vet. Rec..

[30] CNA ‘Makes me not Want to Come Back’: Low Respect, Limited Prospects Drive S’pore Vets to Places like UK, Australia. 27 July 2024. https://www.channelnewsasia.com/today/ground-up/makes-me-not-want-come-back-low-respect-limited-prospects-drive-spore-vets-places-uk-australia-4635086.

[31] The Straits Times Rising Cost of Vet Medicine in S’pore: How Much Do You Need to Have a Pet? 16 September 2024. https://www.straitstimes.com/singapore/rising-cost-of-vet-medicine-how-much-do-you-need-to-have-a-pet.

[32] Scholl I., Kriston L., Dirmaier J., Buchholz A., Härter M. (2012). Development and psychometric properties of the Shared Decision Making Questionnaire--physician version (SDM-Q-Doc). Patient Educ. Couns..

